# Recent advances in developing 3D culture systems of spermatogonial stem cell preservation and differentiation: A narrative review

**DOI:** 10.18502/ijrm.v21i9.14397

**Published:** 2023-10-30

**Authors:** Leyla Fath-Bayati, Leila Naserpour, Mohadeseh Khoshandam, Rahil Jannatifar, Hoda Fazaeli

**Affiliations:** ^1^Department of Tissue Engineering and Applied Cell Sciences, School of Medicine, Qom University of Medical Sciences, Qom, Iran.; ^2^Department of Reproductive Biology, Academic Center for Education, Culture and Research, Qom Branch, Qom, Iran.; ^3^National Institute of Genetic Engineering and Biotechnology (NIGEB), Tehran, Iran.; ^4^Department of Mesenchymal Stem Cells, Academic Center for Education, Culture and Research, Qom Branch, Qom, Iran.

**Keywords:** Tissue engineering, Male infertility, Scaffold, Stem cells, 3D cell culture, Differentiation.

## Abstract

Male infertility has received vast attention in recent years and has no clear etiology in almost 40% of cases. Several methods have been suggested for preserving sperm and spermatogonial stem cells (SSCs) in both in vivo and in vitro conditions. The efficacy of these methods is related to their abilities, including providing an optimal environment for sperm preservation and long-term SSC culture for in vivo and in vitro differentiation of these cells. In this review article, a full MEDLINE/PubMed search was performed using the following search terms: “Spermatogonial Progenitor Cells, Stem Cells, Fertility Preservations, Sperm Freezing, Cell Differentiations, Tissue Scaffold, 3-Dimensional Cell Culture", which retrieved results from 1973-2022. Related articles were added to the bibliography of selected articles. Exclusion criteria included non-English language, abstract only, and unrelated articles. The production of functioning male germ cells is suggested by introducing modern bioengineered systems as a new hope for the maintenance of male fertility. Till now, few in vitro spermatogenesis investigations have provided appreciable amounts of mature gametes. Each method had benefits and disadvantages, but the 3-dimensional culture method had the greatest impact on the differentiation and preservation of SSCs. One of the critical elements of research is the preservation of sperm and the differentiation of SSCs. Several methods have been employed in this area. Various scaffolds providing an environment similar to an extracellular matrix and conditions for germ cell development and survival have been employed in recent research.

## 1. Introduction

Fertility in both men and women has recently attracted attention. Due to continuous low total fertility rates below replacement level (2.1 children per woman), several countries in Asia and the European Union are expected to soon see considerable population declines (1). Some studies revealed a significant decline in semen quality during the past 50 yr (2, 3). Around 40-50% of infertility cases are caused by the “male factor", and up to 2% of males worldwide have sperm with poor quality. As a result, maintaining male fertility is crucial (4). Several disorders and diseases that impact male fertility are more prevalent now than they previously were. The fertility preservation procedure might be required for some systemic diseases or medical therapies that could impair fertility (5).

Non-oncological factors, including genetic disorders, inflammation induced by trauma, infections, and autoimmune diseases, are considered risk factors for male infertility (6-8). Reduced sperm production and function; however, may result from the underlying illness (5). Serious trauma, such as spinal cord injuries linked to erectile and ejaculatory dysfunction, is on the opposite end of the non-oncological spectrum (9, 10). Certain cancers that develop before or after puberty require preserving fertility issues because of chemotherapy and radiation treatments. 80% of cancer survivors are considering starting a family, but only 10% would use donor sperm or adoption.

Vasectomy is one of the other conditions (11), up to 6% of men eventually ask for a reversal when their circumstances and objectives alter (12). In light of this, the patient might be advised about the possibility of sperm freezing before a vasectomy or sperm extraction from epididymis during surgery (13).

Postpubertal males who produce completely developed sperm have the opportunity to have their semen cryopreserved, which allows them to have children later in life through in vitro fertilization (14). Since young boys are unable to produce sperm until puberty, it will be difficult for them to maintain fertility (15). Postpubertal males can be offered a testicular biopsy which contains spermatogonial stem cells (SSCs) (16). SSCs are a subpopulation of type A spermatogonia localized in the seminiferous tubules' basement membrane, and they are essential to the process of spermatogenesis, which produces millions of haploid spermatozoa every day (17). Potentially, isolated SSC or immature testicular tissue can be developed in vitro or returned to the natural physiological microenvironment. Developing SSC niches would make it easier to create a successful long-term SSC culture in vitro, which is the basis for further study and applications (16).

The preservation and culture of germ cells are suggested methods for preserving fertility in these people. In this review, we will focus on the procedure for differentiating and maintaining germ cells and sperm both in vitro and in vivo.

## 2. Materials and Methods

The MEDLINE/PubMed database were thoroughly searched and 6489 articles were found. The following MeSH phrase combinations were used to search “Spermatogonial Progenitor Cells" AND “Stem Cell" AND “Fertility Preservations" AND “Sperm Freezing" AND “Cell Differentiations" AND “Tissue Scaffold" AND “3-Dimensional Cell Culture".

**Figure 1:**
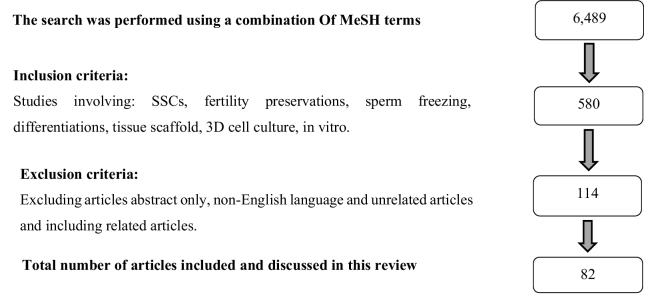
Diagram of article selection.

The exclusion criteria were as: abstract only, non-English language, and unrelated articles. Following the search, it was found that data spanned the time frame of 1973-2022. Relevant articles were added from the bibliographies of selected articles and finally, 82 articles related to the inclusion and exclusion criteria were selected (Figure 1).

All studies, including data related to the development of culture systems, preservation, and differentiation of SSCs, were included in the article. Articles lacking these data were reserved for other qualitative aspects of the article. Finally, only tissue engineering data were extracted from manuscripts that included scaffolds and 3D printing, and SSCs.

### Orgin of SScs

In the postnatal testis, SSCs develop from gonocytes, which grow from primordial germ cells (PGCs) during fetal development (18). In the embryo's epiblast stage, which takes place around 7-7.25 days post coitum (DPC), a tiny group of alkaline phosphatase-positive cells is the first sign of PGCs, a temporary cell population. The extra embryonic ectoderm's expression of bone morphogenetic protein 4 (BMP4) and BMP8b is necessary for PGC specification, and BMP8b is from the extraembryonic ectoderm. When the allantois develops in mice between 8.5 and 12.5 DPC, PGCs start migrating from the hindgut to the indifferent gonad (19).

Due to PGCs proliferation at the migratory phase, approximately 3000 PGCs go through the genital ridges. At 13.5 DPC, PGCs in the male gonad develop into gonocytes, and these gonocytes are enclosed in testicular cords made of Sertoli precursor cells and peritubular myoid cells (20). The terms mitotic (M)-prospermatogonia, T1-prospermatogonia, and T2-prospermatogonia can be used to subcategorize gonocytes.

M-prospermatogonia, located away from the basal membrane in the middle of the testicular cords, continues to expand until 16.5 DPC of mouse development. At this point, they change into T1-prospermatogonia and reach G0 mitotic arrest. During the 1 ${}^{\text{st}}$ wk after birth, gonocytes continue proliferating (signifying their transformation to T2-prospermatogonia), accompanied by migration to the basement membrane of the seminiferous tubules. The first round of spermatogenesis is initiated by T2-prospermatogonia colonizing the basement membrane, making the starting pool of SSCs that support spermatogenesis all through postpubertal life (21). SSCs exist within the basement membrane of the seminiferous tubules of the testis. They have the work of self-renewal and separation into haploid spermatids in grown-ups (22).

For a long time, it was believed that producing sperm cells outside of the original testicular environment in a culture plate was impossible because of the incredibly complex process of spermatogenesis and sperm growth, which happens inside and out over 2 months (21, 23). Modern techniques developed advanced 3-dimensional co-cell cultures, and differentiation of SSC and their precursor cells have, in any case, revolutionized this field. Numerous studies have used embryonic stem cells (ESC) and induced pluripotent stem cells (iPSC) to create male germ cells. Still, it has been found that embryos from studies that start with PGC are normally compared to those produced by studies using ESC or iPSC (24).

Human ESCs and iPSCs have been recognized as significant sources of pluripotent cells for the in vitro production of germ cells. Human iPSCs produced from autologous cells such as skin biopsy-derived fibroblasts, blood cells, or hair keratinocytes have also been used to make germ cells or gonadal support cells (25).

These methods have been used for Klinefelter and nonobstructive azoospermia patients. Besides legal and ethical limitations, an important limitation of human ESC and iPSC-based techniques is the high chance of gathering hereditary and epigenetic alterations during reconstructive procedures (26). Subsequently, human ESC or iPSC induction of germ cells that can transmit hereditary alterations to the sibling, may not be the safest method for preservation. Therefore, the safest method for maturity preservation may not involve human ESC or iPSC generation of germ cells that may pass on genetic material to a relative (27).

### Cellular and molecular pathways in mammalian testis differentiation

The 2 main divisions of the mammalian testicular tissue are the seminiferous tubules and interstitial tissue. It has been demonstrated that the putative testis' tubular structures progressively divide into cords from an amorphous primordium. Seminiferous tubules in young men include various testis cell types and components, including SSCs, basement membrane (deposited by Sertoli cells), peritubular myoid cells, and Sertoli cells. However, in adult males, the SSCs undergo spermatogenesis to differentiate into mature spermatozoa. SSCs are responsible for maintaining the male germ cell pool and can self-renew (28).

At the start of puberty, the Sertoli cells also go through major morphological and functional changes. At stages of development, these supporting cells generate certain types of proteins, cytokines, hormones, and growth factors that ultimately form the blood-testis barrier, playing a crucial role in spermatogenesis and testis formation. Like with all other germ cells, SSCs are in direct contact with Sertoli cells, SSCs and more mature germ cells cannot exist in vivo without Sertoli cells (29). Leydig cells, a distinct type of cell, are also found in the testicular interstitium and respond to luteinizing hormone by producing the steroid hormone (testosterone). This mechanism maintains postpubertal spermatogenesis and begins the androgenic consequences in the male fetus (30).

In primates, there are 2 distinct spermatogonial subtypes. The A pale spermatogonia serves as progenitor cells that can divide to create both A pale and differentiated B spermatogonia, while the A dark spermatogonia are thought to be the testicular stem cell with strong proliferative activity. Incomplete mitosis and further differentiation in spermatogonia result in intermediate and B spermatogonia with cytoplasmic bridges. The maturation of B spermatogonia results in the production of primary and secondary spermatocytes, spermatids, and spermatozoa (29).

After adolescence, additional mitotic and meiotic divisions occur in the second division, which leads to produce primary and secondary spermatocytes and, eventually, spermatozoa. Since 1960, several studies have focused on how to produce meiotic and postmeiotic alterations in male germ cells in vitro. For many years, a major area of study focused on the fundamental principles of male germ cell differentiation, which produces mature haploid spermatozoa inside structurally well-organized tissue. However, according to other investigations, spermatogenesis was stopped before it reached the meiotic phase (31). Another important characteristic of the testis is its dual functions as a glandular and gonad tissue, the in vitro production of sperm from male germline stem cells is still considered difficult because the entire spermatogenic process must occur in a cell culture plate. Also, differentiating SSCs' requires a varied set of paths. The molecular process is crucial for the maintenance and differentiation of testis reproductive cells, which should be considered in vitro culture (32).

Also, molecular pathways are crucial in these cells' differentiation ability. Retinoic acid (RA) and the RA receptor are both necessary for triggering meiosis (33). This stimulates differentiation and blocks factors like promyelocytic leukemia zinc finger (34). The Sertoli cell-secreted stem cell factor can bind to the proto-oncogene receptor tyrosine kinase, which starts a signaling cascade for differentiation (35). The antagonist of *BMP4*, Noggin, prevents RA-induced production of *c-Kit* and *RA-8* (*Stra8*)-stimulated gene expression, but *BMP4* and RA signaling cooperate. Neurogenin 3, whose function is not yet understood, is an intrinsic differentiation element (36). Sertoli cells release glial cell-derived neurotrophic factor (GDNF), and in mice with aging SSCs, GDNF expression is found to be decreased. Fibroblast growth factor 2 (FGF2) is, additionally, important for spermatogenesis (37). Through the extracellular signal-regulated kinase 1/2 and mitogen-activated protein kinase (MAPK) 1/3 pathways, FGF2 and GDNF cause the G1 to S transition. Some transcription factors, including an inhibitor of differentiation 4, LIM-homeodomain proteins, B-cell chronic lymphocytic leukemia/lymphoma 6B, and Ets variant 5, are activated by GDNF stimulation of GDNF family receptor 1 and attributed to spermatogenesis. This is accomplished by activating the rearranged during transfection receptor (38, 39).

**Figure 2:**
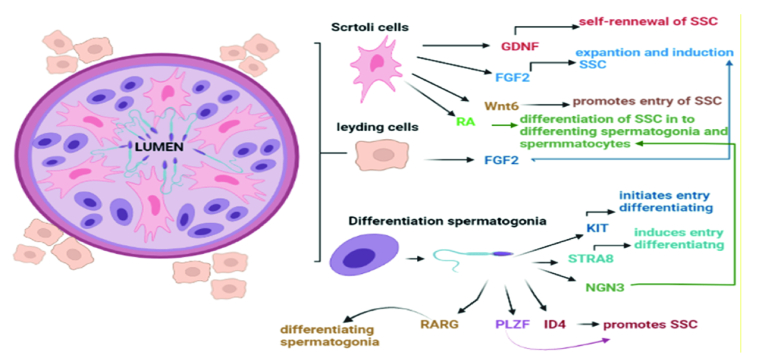
Signaling pathways involved in SSCs differentiation. SSCs: Spermatogonial stem cells, GDNF: Glial cell line-derived neurotrophic factor, FGF2: Fibroblast growth factor 2, Wnt6: Wingless-type MMTV integration site family, member 6, RA: Retinoic acid, KIT: KIT proto-oncogene, receptor tyrosine kinase, STRA8: Stimulated by retinoic acid gene 8, NGN3: Neurogenin-3, ID4: Inhibitor of differentiation protein 4, PLZF: Promyelocytic leukemia zinc finger, RARG: Retinoic acid receptor gamma.

Over the past 10 yr, the major roles of the MAPK, transforming growth factor-/Smad, and MAPKs signaling pathways have been emphasized by increasing evidence in spermatogenesis. The MAPK signaling system controls the dynamics of tight and adherens junctions, meiosis and proliferation of germ cells, and proliferation and lactate production in Sertoli cells, although the transforming growth factor-/Smad and AMPK signaling pathways also have an impact on these dynamics. Together, these signaling pathways create a complicated regulatory system for spermatogenesis (40). A number of the factors that impact differentiation are summarized in figure 2.

### Preservation and differentiation of SSCs

The most advanced treatments for preserving male germline or fertility that are now available include testicular tissue freezing, SSC transplantation, and in vitro spermatogenesis (41). The primary experimental tactics have been emphasized: SSCs transplantation or testicular tissue grafting into host animals and the differentiation of in vitro spermatogenesis. In vitro investigations have focused on preserving germ-cells and producing mature and functional spermatozoa. 2 methods have been suggested for the maintenance of SSCs. Generating viable male germ cells is a new hope for maintaining male fertility that has been made possible by modern bioengineered systems. Future chances for genome preservation and fertility therapy for cancer patients of adult, juvenile, and prepubertal ages, as well as noncancerous infertile males, may be made possible by these procedures (42).

Organ culture systems are considered valuable methods for studying pathophysiological processes because they can effectively represent the functions of the organ in a variety of conditions and environments. The lack of the typical tissue microenvironment can change cellular response (43). The essential cell-cell and cell-matrix signals involved in spermatogenesis are supported by 3D-techniques, which provide all the necessary information about this organ for in vitro spermatogenesis or the maintenance of male fertility (44). By providing better mimics of the species- and age-specific structures of the testis, as well as the biomechanical properties, cell-cell interactions, and natural matrix of the mammalian reproductive tract, 3D cultures that place cells on synthetic matrices, organoids, or organs-on-a-chip cultures, may be able to overcome the limitations of in vitro 2D culture vessels (45). Furthermore, organ cultures allow researchers to control the paracrine environment and separately evaluate the effects of each growth factor on the spermatogenesis process. Many techniques have been developed for the in vitro maintenance and culture of intact tissues. The contact between the seminiferous tubules and the interstitial area may be maintained in the 3D testicular tissue culture systems, which are suitable for the progression of spermatogenesis. To encourage in vitro spermatogenesis; however, such media must be improved by including specific necessary and efficient elements (such as luteinizing hormone, follicle-stimulating hormone, RA, triiodothyronine, testosterone, or other types of antioxidants, vitamins, growth factors, and hormones) (46).

### Testicular organ culture systems

Testicular organ cultures from newborn mice were used in the first study on in vitro spermatogenesis. The first attempts to develop a culture method for testicular tissues were made in 1920. The testicle pieces were preserved in a culture medium based on plasma clot. The plasma clotting method is a culture that may be used to examine the development of fetal organs and testicular tissue (47). The main benefit of this method was to enable the visual study of whole cells with an emphasis on their dynamic properties and shape modulations over time and in response to regularly controlled environmental changes (48).

Another technique is the raft one in which the organ was set on a lens made of paper or rayon to help transfer the tissue. Excess fluid was filtered out, and the net containing the tissue was repositioned on a new pool of medium. A size of around 8 mm is the largest that may be properly maintained. To prevent ripping or crushing of the tissues, great care should be used when dissecting these explants. This approach was utilized to research testis culture and cancer in in vivo models. The target tissue was placed on Trowell's grid system, which was made of perforated steel sheet that is hydrated up to the grid's surface, before being placed in a culture chamber. There is a challenge with the raft floating method. Skeletal tissues are frequently placed directly on the grid due to their higher degree of rigidity, whereas softer tissues, such as the skin or glands, must first be stretched out on rafts to be sustained throughout the grids. This is because different tissues have varying degrees of stiffness. The testis of the adult rat was organs grown using this method. However, the results revealed that most of the tubules had degenerated, with just a tiny number surviving for up to 3 days (49).

Based on a modified plasma clat approach, human epidermal cells were seeded on rafts made of collagen and fibroblasts, and an air-fluid was placed between the cells (50). In cultures of mammalian testicular tissue, degeneration of the germinal cells starts a few days after expansion, followed by complete necrosis of the remaining tissues. Although in some reported signs of seminiferous epithelium differentiation, cultures were not kept in acceptable condition for long enough to comprehensively analyze the outcomes. As a result, substantial efforts were made to create culture methods that would allow testicular germ cells to survive and differentiate (51). Since it is known that the process of in vivo spermatogenesis takes many days to complete, for example, 54 days in a rat, it is essential to choose conditions for long-term maintenance of testicular structure if the spermatogenic process is to be in vitro examined (45).

Stukenborg et al. reported the first successful creation of elongated spermatids in mouse testicular cell aggregates in soft agarose and methylcellulose, and their findings are encouraging (52).

Despite using this technique to produce elongated spermatozoa in mice, the spermatids remained in the pachytene stage. The culture of agars alone is the reason for this lack of development and may be responsible for the low effectiveness of tubulogenesis and spermatogenesis. It is interesting to note that germ cells in the center culture developed SCC aggregates, and were then finally killed due to the lack of support. In a different investigation, neonatal mouse testicles were cultured in agarose for 2 wk at the gas-liquid interphase. Cell aggregates were used to study tubulogenesis, and spermatogonia developed until meiosis (51). Immature mouse testicular tissue was cultured on agarose gels at a concentration of 1.5% (w/v) in 2016 and demonstrated for the first time that morphologically normal spermatozoa with intact acrosomes could be produced in vitro using agarose culture (16). They showed that in this method, in addition to the production of sperms, which are assessed morphologically, histologically, and immunohistochemically, in vitro spermatogenesis could be preserved for more than 2 months. Because of the important role of interaction between Sertoli and SSCs, a 2-layer agarose culture medium was used (53). A 2-layer agarose culture medium was employed by researchers. They demonstrated that colonies forming in the agarose culture's upper layer were free of somatic cells, which were seen at the bottom of the plate. This interaction between spermatogonia and Sertoli cells has also been shown to affect the differentiation of spermatogonia and tubular formation. However, Sertoli cells did not migrate into the bottom layer of the agarose culture in this case because more viscose agarose was present (1.5% vs. 0.5%), preventing Sertoli cells from doing so. Instead, Sertoli cells aggregated in the top layer. According to the immunocytochemistry data, Vimentin, a Sertoli cell marker, was found in aggregations (45).

A study revealed strong modeling of in vitro human testicular organogenesis from the fetal genital ridge, which represents a significant advance for the study of male reproduction. Human gonads from aborted 12-19-wk fetuses were cut up and placed on agarose gel stands. Additionally, the meiotic phases were completed, and the differentiation of spermatogonia was seen morphologically and in terms of gene expression (54).

In vitro-grown supplements containing growth factors or drugs for local or systemic distribution, such as vascular endothelial growth factor, antioxidant compounds, and testosterone, were investigated in recent studies on improving SSCs survival (55).

Several antioxidant chemicals are being researched for their ability to enhance sperm survival, reduce apoptosis, and improve SCC survival (56). Several studies have employed substances like melatonin and n-acetyl cysteine, for example. In contrast to n-acetyl cysteine, which inhibits apoptosis and improves apoptosis and proliferation (57), melatonin affects survival before transplantation but does not affect apoptosis (58).

### Hydrogels method

The extracellular matrix (ECM) mostly comprises proteins and polysaccharides. Laminin is a crucial modulator of protein, paracrine, autocrine growth factor, and Sertoli cell secretion and transfer in the basement membrane, which directly affect the survival and differentiation of testicular cells throughout development (59). One of the fundamental signaling pathways, direct cell-to-cell contact, is crucial for the effectiveness of spermatogenesis and might be efficiently assisted by hydrogels (60). Cells might be implanted in a thick layer using collagen gel matrices, creating an extracellular environment that successfully mimics the activities of the seminiferous tubule basement membrane. Using 2 classes of natural-based polymers, including proteins (like collagen) and polysaccharides (like chitosan and alginate), testicular cells have been ordered spatially in several investigations. The survival of testicular cells is improved by including laminas in the structure of collagen-based gels. In addition to these advantages, such 3D culture microenvironments prevent cell ischemia, particularly in the long run (61). Some of the investigation on 3D scaffold is summarized in table I.

Alginate, collagen, and acellular tissue matrices (produced by decellularizing different tissues or organs) have special characteristics, including the capacity to produce hydrogels with 98-99% aqueous media at physiologic conditions. Testicular cells can develop and migrate more easily on hydrogel testicular matrices. The essential elements of testicular ECM should still be present in these matrices, while cells, bioactive cellular proteins, DNA, and substances that may interfere with the efficiency of culture should be eliminated (49).

Alginate can also lessen the toxicity brought on by freezing when used in cryopreservation. Alginate hydrogels were tested in 2017 for their potential toxicity in the culture systems of SSCs. Surgery was used to extract the SSCs from immature mice that were 6 days old. SSCs were enclosed in alginate hydrogels. The cells were removed from the hydrogel and analyzed for survival, cell shape and structure, cytotoxicity, and expression of the apoptotic genes following encapsulation and cultivation for a month in Dulbecco's Modified Eagle Medium culture media with 10 ng/ml GDNF. Compared to the control group, the main group's expression of the Fas Cell Surface Death Receptor (*FAS*) gene increased, whereas that of the *Bax* and *P53* genes decreased. Cell viability was significantly reduced, but spermatogenesis was restored after the freeze-thawed SSCs encapsulated in alginate hydrogel. Various studies show that alginate hydrogels had high vitality (74.08%) and low cytotoxicity (5%), and protect SSCs from damage (62).

**Table 1:** Some studies on the use of tissue engineering in restoring spermatogenesis


**Author, yr (Ref)**	**3D culture system**	**The aim of the study**	**Results**
**Zhao ** ***et al*** **., 2020 (63)**	Alginate	Evaluation of alginate's effect on spermatogenesis	Alginate could restore busulfan-disrupted spermatogenesis and promote the expression of crucial genes for spermatogenesis
**Baert ** ***et al*** **., 2019 (64)**	Alginate	In vitro*,* spermatogenesis using 3D bio-printed scaffolds based on alginate	There were both elongated and round spermatids present
**Del Vento ** ***et al*** **., 2021 (65)**	Alginate + VEGF	Revascularization of testicular tissue in alginate hydrogel graft or defense against hypoxia injury to cells and tissue	Increased vascular maturation and accelerated vascular structural development in grafts
**Pirnia ** ***et al*** **., 2017 (62)**	Alginate	Evaluating the effect of SSCs cryopreservation in alginate hydrogel on the stemness potential of the cells	Alginate can enhance stemness potential during cell cryopreservation and resume spermatogenesis after transplantation because it resembles the ECM for SSCs
**Hemadi ** ***et al*** **., 2022 (66)**	Alginate	Investigation on the morphology and structure of SSCs enclosed in alginate	The expression of the *Oct4*, *Sox2*, and *Nanos2* genes was reduced in encapsulated SSCs, while the expression level of *Nanog*, *Bcl6b*, and *Plzf* genes was not substantially changed
**Veisi ** ***et al*** **., 2022 (67)**	Alginate	Comparing the effects of co-cultured Sertoli cells and SSCs in a 3D alginate hydrogel	Efficient proliferation and preservation of SSCs stemness and the improvement of SSC transplantation effectiveness can result from the growth of SSCs on alginate hydrogel with Sertoli cells in a 3D culture
**Baert ** ***et al*** **., 2015 (68)**	Human testis	Ascaffold from human testis for the purpose of tissue engineering and regenerative medicine	Generation of a cell-compatible testicular DTM preserving testicular tissue-specific properties and components
**Borzouie ** ***et al*** **., 2020 (69)**	Tricalcium phosphate nanoparticles (TCP NPs) with human serum albumin (3D porous scaffolds)	Utilising cell culture on 3D porous scaffolds, evaluate the proliferation of hTCs	Future applications in regenerative medicine for male infertility might involve the use of 3D HSA/TCP NPs scaffolds to reassemble the artificial human somatic testicular niche
**Borzouie ** ***et al*** **., 2019 (70)**	Nanofibrous scaffolds	A handmade scaffold formed of electrospun nanofibers of homogeneous polyvinyl alcohol/human serum albumin/gelatin (PVA/HSA/gelatin) can be used to cultivate hTCs collected through the TESE technique	hTC growth is supported by the PVA/HSA/gelatin scaffold
SSCs: Spermatogonial stem cells, ECM: Extracellular matrix, VEGF: Vascular endothelial growth factor, *Oct4*: Octamer-binding transcription factor 4, *Sox2*: SRY-box transcription factor 2, *Nanos 2*: Nanos C2HC-type zinc finger 2, *Nanog*: Nanog homeobox, *Bcl6b*: B cell CLL/lymphoma 6, member B, *Plzf*: Promyelocytic leukemia zinc finger, DTM: Decellularized testicular matrix, HAS: Human serum albumin, TCP NPs: Tri-calcium phosphate nanoparticles, hTCs: Human testis-derived cells, TESE: Testicular sperm extraction, PVA: Poly vinyl alcohol, HSA: Human serum albumin			

### Carbon nanotubes method

To nourish, attach, and differentiate spermatogenic cells as well as cure defective spermatogenesis, carbon nanotubes have been effectively created (71). Carbon nanotubes have shown a superb ability to bind serum proteins and encourage cell adhesion, spreading, and development in vitro, even for long-term applications (72). Despite nanocarbon's benefits, cytotoxicity has remained a major cause for worry (which might be reduced by removing serum proteins before cell culture or purifying scaffolds) (73).

### Grafting method

Graft technic is an additional method for sperm storage that obtained full spermatogenesis using tissue from all 3 species in 2002 by grafting tiny fresh biopsies from prepubertal mice, pigs, and goats onto the back skin of nude mice. The study claims that the replacement of segments was just fixed subcutaneously without creating synthetic vascular anastomosis (74).

In the same nude mouse model, the spermatogonial survival rates after orthotopic immature testis tissue (ITT) xenotransplantation decreased over time (61% at 5 days, 14.5% at 3 wk, and 3.7% at 6 months) (75). Moreover, continuing vascularization is necessary for a successful grafting approach because germ cell loss occurs within the first 3 wk. Orthotopic grafting was the optimum choice since studies on germ cell development revealed a detrimental effect of higher temperatures on grafts. Rodent testicular tissue has been implanted in a number of locations, including the back skin, ear tip, the anterior chamber of the eye, and testis. The authors of these data speculate that eliminating the testicular parenchyma could result in a rupture of the integrity of the seminiferous tubules, making it easier for SSCs from the donor to recolonize the area. As a result, intratesticular graft has been suggested as a suitable substitute for intra-scrotal ITT grafting, although being technically more difficult (76).

One study developed an undiscovered in vitro organ culture technique that enables the transplanting of mouse SSC lines which could differentiate into colonies and mature sperm. The haploid cells were recognized by cell morphology through histological and immunohistochemical analysis, and genetic markers (synaptonemal complex protein 1, sperm protein 56) and to achieve fertility, they used micro-insemination, which led to a healthy pregnancy (77).

A spontaneous circulatory connection between the host and the grafted tissue was developed as arteries sprouted from the transplanted component and joined the host's vascular system (49). Since every cell should be placed within at least 200 μm distance of a capillary, any tissue that has a longer distance must have a developed and sustained vascularization to survive, as results in extremely little quantities of testicular tissue ( ± 1 mm^3^) experiencing a period of no blood supply, which is defined by hypoxia, even followed by a condition of reoxygenation, this tissue characterized by damage, as was previously shown for ovarian tissue transplantation (78). In culture-based research, platelet-derived growth factors, FGF, and vascular endothelial growth factors have been used in various studies to increase vascularization. Because of their short half-lives, the dangers of distant dispersion, such as enhanced vascular permeability with the risk of the encouragement of tumor neo-angiogenesis, hypotension, and vascular growth factors present substantial hurdles when employed in vivo. By employing encapsulating matrices and customized nanoparticles, these problems might be solved by the controlled release of physiologically active chemicals contained in the place of interest (79).

### Preservation of fertility in adult males

For adult males and pubertal boys who produce sperm in the ejaculate and who will be receiving gonadotoxic treatment, the cryopreservation of ejaculated semen is advised. Gonadal shielding is a solution if sperm collection is impossible for males receiving radiation therapy (80). The procedure involves the collection of, preferably, 3 semen samples with an abstinence period of at least 48 hr in between samples, followed by the cryopreservation of the sperm samples (81). However, it is frequently necessary to collect more than one semen sample on the same day to prevent delay in oncological treatment. When ejaculation fails or there are no spermatozoa in the ejaculate, collection of the sperm from the epididymis through either percutaneous epididymal sperm aspiration or microsurgical epididymal sperm aspiration can be done (82).

## 3. Conclusion

Spermatogenesis is a susceptible process influenced by endocrine hormones and micro-environmental conditions. Several major illnesses might affect male fertility. These conditions are divided into 2 categories before and after puberty. There is currently no option to preserve the testis tissue except by using various techniques and in vivo or in vitro inducing differentiation in pre-puberty situations when a person does not have mature sperm. In humans, when prepubescent boys face cancer treatment, freezing immature testicular tissue containing SSCs can be suggested. In adulthood, fertility can be restored by autologous tissue transplantation or its SSCs, or in vitro maturation. So far, animal studies have been conducted with good results, but extensive and acceptable clinical trials have not yet been performed. Transplantation of testicular pure cells is impossible due to the risk of reintroducing cancer cells.

Therefore, tissue-engineered testis, can be helpful in achieving spermatogenesis for tissue transplantation or in vitro conditions. In the postpuberty period, sperm can be frozen and used for intracytoplasmic sperm injection under suitable conditions. Many hypotheses suggest that this maturation can be achieved through 2- and 3D tissue culture, and sperm can be obtained. But using this system in different laboratories and providing setup brings various problems. Standardized organoids and organoid scaffolds that can be developed for 3D cultures appear preferable.

In this review, the details of developing various techniques for both mentioned groups have been discussed, we tried to recognize the advantages and disadvantages of various plans and, utilize the best technique for the intended individuals in different conditions. Of course, researchers are still working on determining the best and most efficient strategy, and one of the main issues in this area is how to preserve and differentiate germ cells.

##  Conflict of Interest

The authors declare no conflict of interest.
